# Parent Training for Disruptive Behaviors in Referred Children with Autism Spectrum Disorder: A Randomized Controlled Trial

**DOI:** 10.1007/s10803-024-06567-0

**Published:** 2024-09-27

**Authors:** Simone Breider, Annelies de Bildt, Kirstin Greaves-Lord, Andrea Dietrich, Pieter J. Hoekstra, Barbara J. van den Hoofdakker

**Affiliations:** 1 Department of Child and Adolescent Psychiatry, University Medical Center Groningen, University of Groningen, Groningen, The Netherlands; 2 Accare Child Study Center, Groningen, The Netherlands; 3 The Research School of Behavioural and Cognitive Neurosciences, University of Groningen, Groningen, The Netherlands; 4 Department of Clinical Psychology and Experimental Psychopathology, University of Groningen, Groningen, The Netherlands; 5 Jonx: Department of (Youth) Mental Health and Autism, Lentis Psychiatric Institute, Autism Team Northern-Netherlands, Groningen, The Netherlands; 6 Yulius Organization for Mental Health, Dordrecht, The Netherlands; 7 Department of Child and Adolescent Psychiatry/Psychology, Erasmus MC-Sophia, Rotterdam, The Netherlands

**Keywords:** Parent training, ASD, Disruptive behaviors, Face-to-face, Blended, Online

## Abstract

The purpose of this study was to investigate whether face-to-face and therapist-assisted online (i.e., blended) behavioral parent training are effective on reducing disruptive behaviors in children with autism spectrum disorder (ASD) in routine mental health care. Ninety-seven children with ASD (4–13 years; 76 boys) were randomized to face-to-face parent training, blended parent training, or a waitlist control condition. We assessed treatment effects on parent-rated child noncompliance (primary outcome) and irritability (secondary outcome). This involved comparing both formats separately to the control condition using linear regression models. Child behaviors at 6 months follow-up were also examined. Children in the face-to-face parent training condition improved significantly more on noncompliance and irritability than children in the waitlist condition and improvements sustained to 6 months follow-up. Children in the blended condition did not improve more than children in the waitlist condition and attrition was high. Our results extend findings from efficacy studies to routine mental health care and advocate the use of face-to-face parent training for disruptive behaviors in children with ASD. More research into blended parent training programs for children with ASD and disruptive behaviors in routine mental health care should be conducted to draw more definite conclusions about the value of blended parent training for these children. Trial registration number NL4712; date of registration 22–10–2014.

**Supplementary Information:**

The online version contains supplementary material available at 10.1007/s10803-024-06567-0.

## Introduction

Disruptive behaviors such as noncompliance, irritability, tantrums, and aggression are prevalent in children with autism spectrum disorder (ASD; Kaat & Lecavalier, 2013; Maskey et al., 2013; Presmanes Hill et al., 2014; Stevens et al., 2017) and are associated with child and family burden (Gadow et al., 2008; Herring et al., 2006; Huang et al., 2014; Scahill et al., 2012; Sikora et al., 2013). The evidence for the efficacy of behavioral parent training as a treatment for disruptive behaviors in children with ASD is increasing: two meta-analyses, together including 11 randomized controlled trials, reported that disruptive behavior decreased significantly more in treatment conditions than in control conditions (i.e., mostly care-as-usual or waitlist controls; Postorino et al., 2017; Tarver et al., 2019). In addition, the positive effects of parent training appeared to be maintained 3 to 6 months after parent training (Bearss et al., 2015; Sofronoff et al., 2004; Tellegen & Sanders, 2014; Whittingham et al., 2009). Many of the conducted studies were efficacy studies that recruited participants through school newsletters, websites, and flyers (Kuravackel et al., 2018; Solomon et al., 2008; Tellegen & Sanders, 2014; Zand et al., 2018), offered treatment at university clinics (Aman et al., 2009; Bearss et al., 2015; Handen et al., 2015), or provided parent training by students (Ginn et al., 2017; Sofronoff et al., 2004; Whittingham et al., 2009). However, so far, effectiveness studies, conducted in routine mental health care, remain scarce. Investigating the value of parent training in routine mental health care is important to improve the ecological validity and enhance their generalization to clinical settings (Fahmie et al., 2023).

Besides the focus on non-clinical samples and settings, the evidence for parent training in children with ASD is largely based on face-to-face parenting programs (Postorino et al., 2017; Tarver et al., 2019). However, it has been demonstrated that practical barriers, such as a demanding daily life, a large distance to a treatment provider, and long waiting lists can hamper the start and continuation of this parent training format (Koerting et al., 2013; Mytton et al., 2014; Raulston et al., 2019). Online parent training, in which parents progress through a digital training program, with or without therapist assistance, could possibly address these barriers. Advantages of online programs may be that they are more flexible and easier to implement in parents’ daily lives, as well as less time-consuming for therapists and thus less costly (Enebrink et al., 2012). Furthermore, they may be a good alternative when providing training at a treatment location is not possible, for example during the COVID-19 pandemic (Sullivan et al., 2021). Since the pandemic, remote methods have been used more and more often in care for ASD, for instance to provide direct one on one instruction to students with ASD (e.g., Henry et al., 2023 for listening comprehension; Kahng et al., 2023 for job interview skills) and therapist training (e.g., Lloveras et al., 2022 for conducting functional analyses). Additionally, it has been demonstrated that online parent training can reduce disruptive behaviors in general population samples and in children with attention-deficit/hyperactivity disorder (ADHD; Baumel et al., 2016; DuPaul et al., 2018; Thongseiratch et al., 2020), and that online parent training can improve other problems than disruptive behavior in children with ASD (i.e., parent–child interaction, daily routines, social communication, and sleep problems; Blackman et al., 2020; Ibañez et al., 2018; Ingersoll et al., 2016; Ip et al., 2024). However, its effect on disruptive behaviors in children with ASD needs to be investigated. Furthermore, since multiple studies have shown that therapist assistance can positively affect outcomes and acceptability of online parenting interventions (Day & Sanders, 2018; Ingersoll et al., 2016; Tarver et al., 2014), research into such blended parent training is valuable. More generally, more knowledge is needed on the effectiveness of online treatment for externalizing behaviors (Ros-DeMarize et al., 2021).

In the current randomized controlled trial, we investigated the effectiveness of behavioral face-to-face as well as blended parent training on the reduction of disruptive behaviors in children with ASD in routine mental health care. This involved comparing both formats separately to the control condition. Because this was the first time that each of the formats of parent training was studied in routine mental health care, and research into online parent training for children with ASD and disruptive behaviors is scarce altogether, we did not aim to compare both formats to each other. Our primary outcome was child noncompliance, while we secondarily focused on child irritability. Both parent training formats were developed from a face-to-face parent training program that was shown to reduce disruptive behaviors in children with ADHD (Van den Hoofdakker et al., 2007; Van der Veen-Mulders et al., 2018). We hypothesized that disruptive behaviors would reduce significantly more in children of parents receiving (either format of) parent training, than in children in the waitlist control condition. Since previous studies showed that age may influence the effect of parent training (Daley et al., 2014; Farmer et al., 2012), we included child’s age as a covariate in our analyses. To explore long-term changes in disruptive behaviors, we compared the severity of disruptive behaviors directly after each training to that 6 months after the training. Finally, we exploratorily investigated the effects of site and several variables that are available in clinical practice (i.e., sex, IQ, ASD severity, and parental education) on the reduction of disruptive behaviors. The few previous studies into predictors and moderators of parent training effects in children with ASD were conducted outside of routine mental health care. These reported only few factors to be associated with treatment effect (Farmer et al., 2012; Lecavalier et al., 2017), indicating that a broad range of children with ASD may benefit from parent training. We explored this in routine mental health care in our study.

## Methods

### Participants

Participants were 97 children with a clinical diagnosis of ASD and co-occurring parent-reported disruptive behaviors, recruited from two outpatient centers for child and adolescent psychiatry in the Netherlands. Inclusion criteria regarding the child were: (1) diagnosis of ASD according to the DSM-IV-TR (American Psychiatric Association, 2000) or DSM-5 (American Psychiatric Association, 2013), based on consensus after diagnostic assessments [including semi-structured interviews and developmental history and observation of the child, mostly using the Autism Diagnostic Observation Schedule-2 (De Bildt et al., 2013; Lord et al., 2012)] in a multidisciplinary team specialized in ASD, with at least a psychologist with post-master degree, trained and licensed for diagnosing ASD and a child and adolescent psychiatrist, also trained and licensed for diagnosing ASD; (2) age between 4 and 13 years, i.e., in line with the Dutch primary school age range; (3) total IQ higher than 50, as assessed with the Dutch version of the Wechsler Preschool and Primary Scale of Intelligence III (Hendriksen & Hurks, 2011) or the Wechsler Intelligence Scale for Children III (Kort et al., 2005); (4) no psychotropic medication use, or being on a stable dose for at least 6 weeks before study entry, meaning that the prescribing psychiatrist was not expecting changes in dose or agent at that time; and, (5) at least three disruptive behaviors at home, as reported by at least one parent on a list of 32 target behaviors (e.g., noncompliance, arguing, temper tantrums, claiming attention [see Supplementary file 1 for the complete list (Van den Hoofdakker et al., 2007)].

In addition, inclusion criteria regarding the parents were: (6) at least one parent was willing to participate in the parent training; and (7) parents had a laptop or PC at their disposal. Exclusion criteria were: (1) parents had participated in behavioral parent training in the year prior to the study (based on parent report); (2) the family required immediate intervention (e.g., families in which one of the parents was experiencing acute mental problems or in which the safety of the child could not be guaranteed); and, (3) the family had plans to move to a region far from one of the study locations within 6 months. No restrictions were applied to children’s comorbid mental disorders.

### Study Design, Procedures, and Sample Size Calculation

The study was a two-center randomized controlled trial with two intervention conditions (face-to-face parent training and blended parent training) and a 20-week parent training waitlist condition. Both parent training conditions were compared to the waitlist condition, but not to each other. In all three conditions, children and parents were allowed to receive other (mental) health care, except behavioral parenting interventions directed at the child’s behavior. Inclusion took place from December 2014 to March 2018. The trial was approved by the Medical Ethics Review Committee of the University Medical Center Groningen.

Parents of potentially eligible children were asked by their clinician to participate in the study and give consent to receive a phone call from a researcher. If consent was given, this researcher informed parents and children in more detail about the study and evaluated eligibility. Parents and children aged 12 signed informed consent when they agreed to participate.

**Fig. 1:**
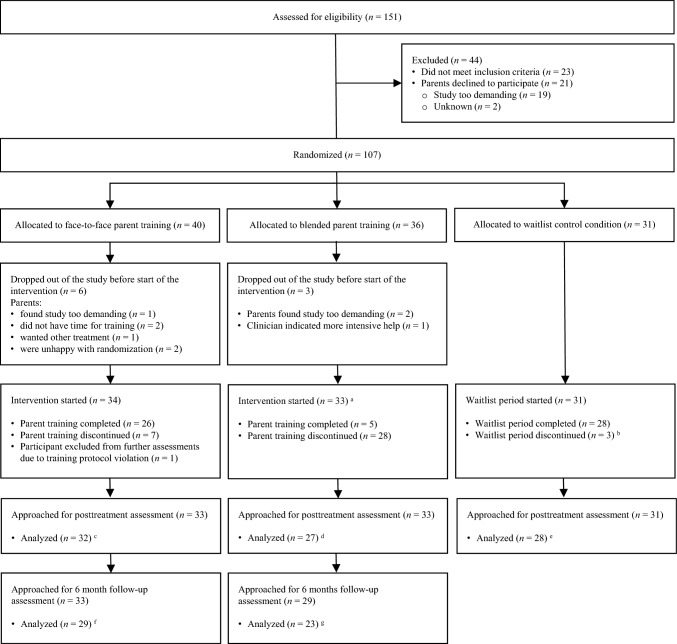
Study flow chart. ‘Start’ of the intervention signified parental attendance of the first session of parent training. See results section for information on (reasons for) discontinuation of parent training. **a** In one participant, medication was stable for 4 instead of 6 weeks. **b** Parents of two children discontinued because they (after 6 and 13 weeks respectively) did not want to wait any longer before receiving parent training. For one child, the waitlist period was discontinued after 6 weeks because immediate care was indicated by the clinician due to severe worsening of problems. Participants whose data was missing concerned: **c** One treatment dropout, **d** Six treatment dropouts, **e** Three waitlist completers, **f** Three treatment dropouts and one completer, **g** 10 treatment dropouts

Figure 1 displays the flow of participants throughout the study.

After baseline assessments, an independent research assistant randomized participants with a random number generator, separately for each of the two outpatient centers. Of the final sample of 97 participants, 33 were allocated to face-to-face parent training, 33 to blended parent training, and 31 to the waitlist condition. Participants were approached for posttreatment assessment if they had started one of the three study conditions; that is, they were also approached if they discontinued parent training or the waitlist period prematurely (see Fig. 1 for information on discontinuation). Posttreatment assessment took place directly after the parent training or the waitlist period stopped.

We estimated a required sample size of 118 (i.e., 40 in both parent training conditions and 38 in the waitlist condition), for an 80% power to detect an effect of parent training (both formats) versus the waitlist condition with an effect size of 0.65 on our primary outcome measure, i.e., the Home Situations Questionnaire—Pervasive Developmental Disorders (HSQ-PDD; Aman et al., 2009). Our estimation was based on Aman et al. (2009) and Solomon et al. (2008).

The trial has been registered in the Dutch Trial Register (see https://onderzoekmetmensen.nl/en/trial/22042).

### Interventions

#### Face-to-Face and Blended Behavioral Parent Training

Table 1 provides an overview of the four phases and the topics of the face-to-face and blended behavioral parent training formats.

**Table 1:** Overview of phases and topics in the face-to-face and blended parent training

Phase	Face-to-face parent training		Blended parent training	
	Session	Description of session	Module	Description of module part
1. Introduction and psychoeducation	1	Introduction	1	Introduction (face–to-face)
	2	Psychoeducation	2	Psychoeducation
	3	ABC charts	3	ABC charts
	4	Recording observation of behavior		Recording observation of behavior, impeding factors
	5	Analyzing video, impeding factors		
			Evaluation (face-to-face)	
2. Techniques to manipulate antecedents of behavior	6	Offering structure	4	Communication (instructions and anticipating misbehaviors)
	7	Communication, instructions		
	8	Communication, anticipating misbehaviors		Setting rules
				Offering structure
	9	Setting rules		
	10	Phase 2 recap ^a^		
			Evaluation (face-to-face)	
3. Techniques to manipulate consequences of behavior	11	Rewarding	5	Rewarding
	12	Ignoring and punishing		Ignoring
				Punishing
				Evaluation (face-to-face)^a^
	13	Reward system Part 1^a^		Reward system Part 1^a^
	14	Reward system Part 2^a^		Reward system Part 2^a^
	15	Reward system Part 3^a^		Reward system Part 3^a^
	16	Time out^a^		Time out (face-to-face)^a^
4. Evaluation and generalization	17	Evaluation	6	Evaluation (face-to-face)
	Follow-up after 3 months^b^		Follow-up after 3 months (face-to-face)^b^	

*ABC* antecedent behavior consequence

^a^Optional training topic that could be added to the mandatory parts if needed

^b^Post-treatment assessment took place before the follow-up session after 3 months

At the start of the training, parents selected three to five problem behaviors from the same list of target behaviors that was used to assess disruptive behaviors at inclusion (see Supplementary file 1; Van den Hoofdakker et al., 2007). Parents also selected three to five situations from the HSQ-PDD (Aman et al., 2009). These selected behaviors and situations were targeted during the parent training. The training consisted of mandatory and optional parts, see Table 1.

#### Specifics of Face-to-Face Parent Training

The face-to-face behavioral parent training program was a manualized individual training program for parents of children with ASD and disruptive behaviors. For the study a research protocol was used, which was published for clinical use after the study (Van Warners et al., 2020). The office-based sessions, all provided by the same therapist, lasted between 45 and 60 min. The sessions followed the same structure, generally starting with a discussion of homework assignments, followed by the introduction of a new topic, exercises to help parents understand and practice with that topic, and finally, a new homework assignment for the new topic. Homework for parents between sessions included practicing behavioral techniques and improving understanding by reading chapters of a book on parenting techniques (Van der Veen-Mulders et al., 2010). Each therapist was advised to schedule the first five sessions on a weekly basis and successive sessions bi-weekly. The therapist could add extra sessions if parents did not fully comprehend topics or techniques.

#### Specifics of Blended Parent Training

The blended behavioral parent training included an online training program with six modules in which parents were provided with written theory and exercises, based on the face-to-face parent training program. It started with a 90-min office-based face-to-face contact with the therapist, in which target behaviors and situations were selected and parents were familiarized with the online program. The main content of the training was delivered through the online program. Parents progressed through the different training parts in a set order (see Table 1). Each training part included multiple exercises and reading assignments, similar to the exercises and assignments in the face-to-face training. These included completing an antecedent behavior consequence (ABC)-chart, answering questions about parents’ parenting practices, practicing with a behavioral technique, reading theoretical parts, and informing the therapist about practicing (by writing in the program). The therapist was instructed to provide online written feedback on every exercise and give parents access to the next training part as soon as they had finished the previous topic. In addition, the training included at least two intermediate 45 to 60-min office-based face-to-face contacts to evaluate parental progress (see Table 1). If the therapist noticed that parents had difficulty understanding a topic, they could provide additional office-based face-to-face contacts to clarify the online content. The treatment manual instructed the therapist to remind parents if there had been no activity for more than 2 weeks, and to finish the training within 20 weeks.

#### Therapists and Treatment Fidelity

Parent training was delivered by 42 therapists from the outpatient mental health centers. They were psychologists (at least post-master level) and/or cognitive behavior therapists (post-master or university of applied sciences level) who had received training in the face-to-face and blended parent training (i.e., 2 days per format) from board-certified cognitive behavioral therapists. Therapists without post-master education in cognitive behavioral therapy received monthly supervision by an experienced psychologist with post-master education in cognitive behavioral therapy. Therapists provided parent training based on availability; 41% delivered both formats, 33% only face-to-face, and 26% only blended.

Treatment fidelity was estimated with a treatment integrity checklist. After each face-to-face session, the therapist self-rated whether they had addressed each section (e.g., performing a specific exercise, teaching a new behavioral skill). Therapists who provided blended parent training also self-rated this for each face-to-face session and they self-rated for each online assignment whether they had given feedback on parents’ input.

### Measures

The primary parent (i.e., the parent who spent most time with the child) completed the measurements. For all but one participant, the primary parent attended the parent training, either alone or together with a secondary parent. For one participant in the blended condition, measurements rated by the secondary parent were used, since this parent attended the parent training alone.

#### Primary Outcome

Our primary outcome measure was the HSQ-PDD (Aman et al., 2009), a -modified version of the 20-item Home Situations Questionnaire (Barkley et al., 1999) on which parents rated the occurrence of child noncompliance in 25 everyday situations (e.g., playing alone, at bedtime, during an unexpected change in daily routine) and its severity on a 9-point Likert scale [range 0 (absent) – 9 (severe)]. This modified version (the HSQ-PDD) was developed by investigators of the Research Unit on Pediatric Psychopharmacology (RUPP) Autism Network by adding five situations that were relevant for children with ASD (Chowdhury et al., 2010). The HSQ-PDD has shown to be a valid instrument to measure noncompliance in children with ASD (Chowdhury et al., 2010) and has been used in studies on a parent training program similar to the one in the current study (Aman et al., 2009; Bearss et al., 2015). We used the mean score (Cronbach’s α = 0.92 at baseline).

#### Secondary Outcome

We used the Irritability subscale of the parent-rated Aberrant Behavior Checklist (ABC; Aman et al., 1985) to measure child irritability. The subscale consists of 15 items (e.g., temper tantrums, aggressive to other children or adults, irritable and whiny) rated on a 4-point Likert-scale (range 0–45 with higher scores indicating more irritability, α = 0.86 at baseline). In a large sample of youth with ASD (Norris et al., 2019), the Irritability subscale was shown to have excellent internal consistency and support for convergent, divergent and factor validity was found. The ABC Irritability subscale has been used as measure of disruptive behavior in similar parent training studies (Aman et al., 2009; Bearss et al., 2015).

#### Demographics

At baseline, information was collected on child intelligence, ASD severity, and child and family characteristics, i.e., child age, child sex, comorbidity, marital status, and parental education. ASD severity was assessed with the calibrated severity score on the Autism Diagnostic Observation Scale (ADOS-2) with a range from 1 (minimal to no symptoms) to 10 (high level of symptoms; De Bildt et al., 2013; Lord et al., 2012).

In addition, information on comorbid child mental disorders, mental health care utilization (other than parent training), and the child’s medication use was collected from medical files (collected until discontinuation of parent training/waitlist period). At baseline, post-treatment, and follow-up assessment, parents also reported which mental health care and medication their child had received.

### Data Analyses

#### Baseline Characteristics

To test whether participants in the three conditions differed with regard to baseline characteristics, analyses of variance or Chi-square tests were applied as appropriate. When significant differences were found in baseline characteristics, pairwise post-hoc comparisons were conducted. Alpha level of 0.05 was not adjusted since the baseline tests concerned individual tests, and post-hoc comparisons were restricted to a small number of planned comparisons (Armstrong, 2014).

#### Baseline to Post-treatment Effects: Main Analyses

To test the effects of both parent training formats on child disruptive behaviors, we conducted separate linear regression analyses for the HSQ-PDD (primary outcome measure; model 1) and ABC Irritability subscale (secondary outcome measure; model 2). For both models, the dependent variable was the difference between the baseline and post-treatment scores, with a higher difference score indicating more improvement. In both models we compared each parent training format to the waitlist condition separately, by including a ‘face-to-face/waitlist’ and a ‘blended/waitlist’ dummy variable in the models (parent training coded as 1 and waitlist as 0). The parent training formats were not compared to each other. All participants with post-treatment assessment were included in the analyses, thus irrespective of parents completing or prematurely discontinuing parent training.

Age was included in both models as a covariate. In addition, to examine and control for the influence of baseline severity of disruptive behaviors, we included the scores on the respective outcome measures at baseline as covariates (Twisk et al., 2018). For age as well as baseline severity, we tested the assumption of homogeneity of regression slopes by including the interaction terms of these variables with both condition dummies. Importantly, if an interaction was significant, the interaction terms were kept in the model and the variable was centralized.

Furthermore, we tested the assumptions of normality of residuals, homogeneity of variance, and linearity of continuous predictors (i.e., baseline outcome measure and age, using partial plots), explored outliers (i.e., cooks distance < 1), and multicollinearity (i.e., variance inflation factor < 4).

Effect sizes were calculated by taking the difference in mean change from baseline to post-treatment assessment between a parent training format and the waitlist control condition, and dividing it by the pooled standard deviation at baseline, using a bias correction (Morris, 2008).

#### Baseline to Post-treatment Effects: Exploratory Analyses

We explored whether the child’s sex, IQ, ASD severity, parental education, and site affected the effects of the interventions by adding them, one-by-one, as a main effect and subsequently as interaction effects (with both condition dummies) to the HSQ-PDD and ABC Irritability models. In addition, to inform clinical care, we calculated the number of participants who had improved at least 25% on the HSQ-PDD (the primary outcome measure), and tested differences in these numbers between the three conditions by means of chi-square tests, using HSQ-PDD values corrected for the model 1 covariates HSQ-PDD baseline and age. Finally, to examine if there were differences between the three conditions in use of additional health care or change in child medication use, chi-square tests were conducted.

#### Post-treatment to 6 Months Follow-Up Analyses

To explore long-term changes in both parent training conditions, we used a paired *t*-test per condition to examine the severity of child problem behavior (HSQ-PDD and ABC Irritability) at 6 months follow-up in comparison to post-treatment measurement. This analysis was thus done within each treatment group and should be seen as exploratory because a waitlist condition group was no longer available at 6 months follow-up. Because participants could freely change treatment or medication after parent training, we conducted sensitivity analyses in which we excluded participants of whom health care had changed between post-treatment measurement and 6 months follow-up.

All analyses were carried out with IBM SPSS Statistics (v25), using an alpha level of 0.05 for all tests.

## Results

### Participant Characteristics

Baseline characteristics of the participants in the three conditions are displayed in Table 2.

**Table 2:** Baseline characteristics by intervention condition and analyses of differences

	Face-to-face parent training (*n* = 33)		Blended parent training (*n* = 33)		Waitlist control (*n* = 31)		Differences between conditions	*p*
Child characteristics								
Age: mean (*SD*)	7.97	(2.30)	7.94	(2.25)	7.23	(2.08)	*F* = 1.15	.32
Male sex: *n* (%)	21	(63.6)	26	(78.8)	29	(93.5)	χ^2^ = 8.44	.02^a^
IQ: mean (*SD*)^b^	97.5	(16.9)	99.6	(17.2)	97.7	(15.5)	*F* = 0.16	.85
ADOS-2 comparison score: mean (*SD*)^c^	5.44	(1.88)	5.41	(2.30)	5.48	(1.88)	*F* = 0.01	.99
HSQ-PDD: mean (*SD*)	2.94	(1.66)	3.12	(1.64)	3.02	(1.63)	*F* = 0.10	.90
ABC Irritability: mean (*SD*)	10.2	(6.22)	12.5	(8.21)	10.7	(6.76)	*F* = 0.96	.39
Comorbid diagnoses: *n* (%)^d^							χ^2^ = 3.47	.48
No comorbid diagnoses	24	(77.4)	26	(78.8)	23	(74.2)		
ADHD	4	(12.9)	2	(6.1)	6	(19.4)		
Other	3	(9.7)	5	(15.2)	2	(6.5)		
Child takes psychotropic medication: *n* (%)^e^	5	(15.2)	3	(9.1)	6	(19.4)	χ^2^ = 1.38	.50
Ongoing mental health care for child at inclusion: *n* (%)^f^							χ^2^ = 2.92	.82
Directed at parents	1	(3.0)	1	(3.0)	2	(6.5)		
Directed at child	2	(6.1)	6	(18.2)	4	(12.9)		
Directed at parents and child	1	(3.0)	1	(3.0)	1	(3.2)		
Parent characteristics								
Single parent family: *n* (%)	4	(12.1)	7	(21.2)	2	(13.4)	χ^2^ = 3.07	.22
Educational level: *n* (%) ^g^							χ^2^ = 1.52	.82
Low	2	(6.1)	1	(3.0)	1	(3.2)		
Middle	14	(42.4)	18	(54.5)	17	(54.8)		
High	17	(51.5)	14	(42.4)	13	(41.9)		

^a^Post-hoc comparisons showed: waitlist > face-to-face (χ^2^ = 8.37; *p* = .004)

^b^IQ: *n* face-to-face parent training = 31; *n* blended parent training = 32; *n* waitlist = 30

^c^ADOS-2: *n* face-to-face parent training = 32; *n* blended parent training = 32; *n* waitlist = 31; 75% of children received an ADOS-ASD or ADOS-autism classification

^d^Comorbid diagnoses: *n* face-to-face parent training = 31; *n* blended parent training = 33; *n* waitlist = 31; note on ADHD: one child in the blended group was also diagnosed with oppositional defiant disorder; other = language disorder (three in blended group), developmental coordination disorder (one in face-to-face group), elimination disorder (two in face-to-face group), feeding disorder (one in blended and one in waitlist group), mental retardation (one in blended and one in waitlist group)

^e^Medication concerned psychostimulants (five in the face-to-face group, of which one child also took a benzodiazepine and one an anti-epileptic agent; one in the blended group; and five in the waitlist group) and risperidone (one in the blended and one in the waitlist group)

^f^Health care directed at parents concerned supportive counseling for parents (no parent training); health care directed at the child concerned for example behavioral therapy, creative therapy, social skills training, help at school or a buddy

^g^Concerns the educational level of the parent with the highest educational level in the household. Low = no education, primary school, lower vocational and lower secondary education; middle = intermediate and higher secondary education; high = higher education

*ADOS* Autism Diagnostic Observation Scale, *HSQ-PDD* Home Situation Questionnaire-Pervasive Developmental Disorders, *ABC* aberrant behavior checklist; *n* center 1 = 91; *n* center 2 = 6

Due to difficulty in achieving the aimed number of participants and despite extending the planned inclusion period, inclusion was terminated after 97 participants (82% of the 118 planned participants). Reasons for this difficulty included parents not wanting to be randomized, parents feeling that the parent training would be too time-intensive or that they could manage disruptive behavior without help, and clinicians offering other care than parent training (e.g., medication, treatment at home). The total sample had a mean age of 7.72 years (*SD* = 2.22, range: 4–12) and 78.4% of the children were male. The waitlist control group included significantly more males than the face-to-face parent training group. The other baseline characteristics did not differ between conditions.

### Baseline to Post-treatment Effects: Primary and Secondary Outcome

There was a significant difference of HSQ-PDD improvement between the face-to-face parent training and waitlist condition. The effect size of the face-to-face parent training effect on the HSQ-PDD score was 0.53 [95% CI (0.05, 1.02)]. There was no significant difference of HSQ-PDD improvement between the blended parent training and waitlist condition. Table 3 displays the results of the regression analyses.

**Table 3:** Regression analyses testing the difference in improvement from baseline to post-treatment of child noncompliance (HSQ-PDD) and irritability (ABC Irritability) between each parent training condition and the waitlist condition

	b	*SE*_b_	*b*	*t*	*p*
HSQ-PDD (*n* = 87)					
Constant	− 0.66	0.33		− 1.98	.05
Condition (BPT-F vs. wl)	0.72	0.31	0.24	2.28	.03
Condition (BPT-B vs. wl)	0.32	0.33	0.10	0.97	.33
HSQ-PDD baseline	0.44	0.08	0.50	5.63	< .001
Age (centralized)	0.34	0.11	0.51	3.11	.003
Age * Condition (BPT-F vs. wl)^a^	− 0.31	0.14	− 0.30	− 2.20	.03
Age * Condition (BPT-B vs. wl)^a^	− 0.16	0.16	− 0.12	− 0.97	.34
ABC Irritability (*n* = 86)					
Constant	− 7.99	2.06		− 3.88	< .001
Condition (BPT-F vs. wl)	3.01	1.19	0.28	2.52	.01
Condition (BPT-B vs. wl)	2.01	1.28	0.18	1.57	.12
ABC Irritability baseline	0.25	0.07	0.33	3.46	.001
Age	0.74	0.24	0.30	3.12	.001

^a^Interaction terms between age and the condition dummies were included since the assumption of homogeneity of regression slopes was violated for age; age was centralized at a mean of 7.69

*BPT-F* face-to-face parent training, *BPT-B* blended parent training, *wl*. Waitlist, *vs.* versus

Regarding the covariate age, the regression analyses showed a significant interaction effect between age and treatment condition (i.e., face-to-face versus waitlist), indicating that the child’s age affected HSQ-PDD improvement significantly more in the waitlist condition than in the face-to-face condition. The figure in Supplementary file 2 shows that especially younger children in the face-to-face parent training condition improved in comparison to the waitlist condition. Furthermore, the figure shows that in the waitlist condition, younger children improved less than older children and some even deteriorated. In the face-to-face group, the level of improvement in HSQ-PDD scores appeared similar across ages. Because the boxplot in Supplementary file 2 showed a few outliers, we performed a sensitivity analysis on our HSQ-PDD model without these outliers. Although the outcome was similar in direction, the interaction effect between age and treatment condition (i.e., face-to-face versus waitlist) no longer reached significance (*p* = 0.058). Excluding the age interaction terms from the model showed a significant effect of face-to-face parent training, no effect of blended parent training, and a significant positive effect of age altogether.

**Fig. 2:**
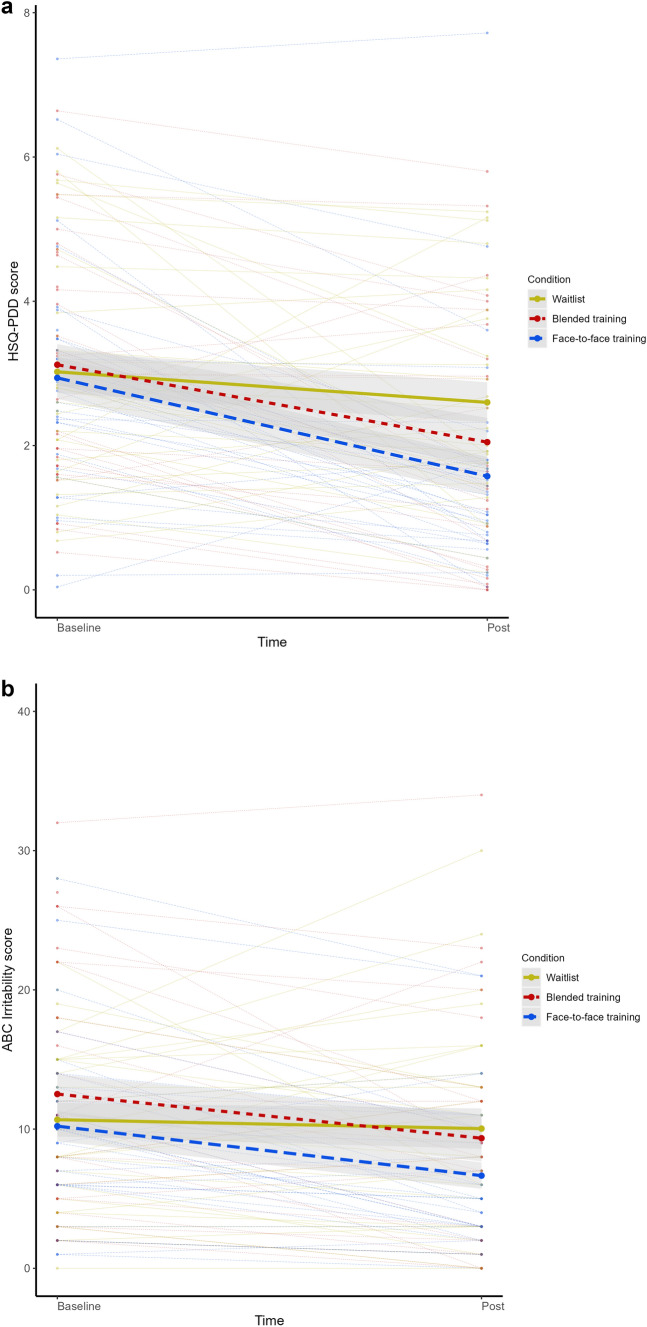
**a** HSQ-PDD and **b** ABC Irritability plot of mean trajectories per condition with confidence bands and individual trajectories (raw data)

The regression analyses showed a significant effect of the covariate HSQ-PDD score at baseline on HSQ-PDD improvement across the three conditions: the higher the HSQ-PDD score at baseline, the more improvement (0.44 for each additional point on HSQ-PDD at baseline). Of note, this was the same in the sensitivity analyses described above. Figure 2 shows a plot of mean trajectories per condition and individual trajectories from baseline to post-treatment (raw data) for each outcome measure, see Fig. 2a for HSQ-PDD. We refer the reader to Supplementary file 3 for plots displaying the outcomes of both regression models, showing mean score per condition at baseline and post-treatment, corrected for covariates in the model (i.e., estimated marginal means), see Supplementary file 3a for HSQ-PDD.

Regarding ABC Irritability, we found that children of parents who had received face-to-face parent training improved significantly more than children in the waitlist group [see Table 3, Fig. 2b, and Supplementary file 3b; effect size 0.62 (0.24, 1.00)]. There was no significant difference between the blended parent training and waitlist condition. The covariates age and baseline irritability had a significant positive effect on improvement over time across the three conditions, i.e., older children and children with higher baseline Irritability scores improved more. There were no significant interaction effects.

### Baseline to Post-treatment Effects: Exploratory Analyses

No effects were found of child sex, IQ, ASD severity, parental education, and site on HSQ-PDD improvement, nor on ABC Irritability improvement.

The percentage of participants who had improved at least 25% over time on the HSQ-PDD was 78.1% in the face-to-face condition, 55.6% in the blended condition and 42.9% in the waitlist condition (χ^2^ = 8.01, *p* = 0.02), see Table 4. Improvement differed significantly between the face-to-face and waitlist condition (χ^2^ = 7.86; *p* = 0.005). It did not differ significantly between the blended and waitlist condition (χ^2^ = 2.88; *p* = 0.09).

**Table 4:** Number and percentage of participants per condition who improved at least 25% on the HSQ-PDD

	Improved	
	n	*%*
Face-to-face (n = 32)	25	78.1
Blended (n = 27)	15	55.6
Waitlist (n = 28)	12	42.9

Face-to-face > Waitlist (χ^2^ = 7.86; *p* = .005); blended versus waitlist (χ^2^ = 2.88; *p* = .09)

There were no differences between conditions in use of additional mental health care (χ^2^ = 0.15, *p* = 0.93) or change in medication use (χ^2^ = 11.8, *p* = 0.07): approximately two-thirds of participants used additional health care (e.g., psycho-education, parent support, child training, help at school) and most participants did not use medication.

### Child Outcome Measures: 6 Months Follow-Up

Table 5 shows the mean raw scores on the HSQ-PDD and ABC Irritability at baseline, post-treatment, and 6 months follow-up in the two parent training conditions.

**Table 5:** Uncorrected means and standard deviations for child outcome measures at baseline, post-treatment, and 6 months follow-up for each condition for participants with a baseline and post-treatment score, and tests of post-treatment to follow-up differences using paired *t*-tests within each parent training condition to evaluate longer term effects

	Baseline			Post-treatment			6 months follow-up			Test of post-treatment to follow-up differences			
	*M*	*SD*	*n*	*M*	*SD*	*n*	*M*	*SD*	*n*	Mean dif.^a^	*SD*	*t*	*p*
HSQ-PDD													
Face-to-face	2.92	1.69	32	1.58	1.50	32	1.32	1.37	29	0.27	1.42	1.02	.32
Blended	3.15	1.65	27	2.05	1.75	27	2.02	1.89	23	0.02	0.68	0.15	.88
Waitlist	3.03	1.71	28	2.60	1.47	28							
ABC Irritability													
Face-to-face	10.1	6.29	32	6.66	5.34	32	5.61	5.01	28	0.96	5.16	0.99	.33
Blended	12.5	8.48	26	9.35	8.46	26	7.86	8.38	22	1.32	3.06	2.02	.06
Waitlist	9.61	5.91	28	10.0	7.65	28							

^a^Mean difference between post-treatment and follow-up measurement, i.e., post minus follow-up

Furthermore, it shows the results of paired *t*-test analyses testing post-treatment to follow-up differences within each condition. In both conditions, there were no significant differences between post-treatment and follow-up on the HSQ-PDD and ABC Irritability. HSQ-PDD and ABC Irritability scores did thus not significantly decrease or increase between post-treatment and follow-up.

During the 6 months follow-up period, approximately one-third of participants in the face-to-face and blended conditions received mental health care such as child therapy, intensive family treatment, or additional parent training. The majority of participants did not change with regard to psychotropic medication use. Sensitivity analyses showed that exclusion of participants who had received mental health care during the follow-up period or for whom medication had changed, did not affect the outcomes of the paired *t*-tests for HSQ-PDD or ABC Irritability.

### Post hoc Analyses

To better understand our finding that children in the face-to-face parent training condition improved on disruptive behaviors in comparison to the control group, but children in the blended condition did not, we compared the two parent training conditions on a number of possibly relevant variables: self-rated therapist fidelity, the number of parents who discontinued the parent training prematurely, the reason for discontinuation, and the number of extra face-to-face sessions that were added if parents did not comprehend topics. Finally, we also explored the relationship between treatment dosage and improvement on the outcome measures.

The reason for discontinuation was inferred from information provided by the therapist and/or from information parents provided in the post-treatment questionnaire or during the phone call in which they were invited to the post-treatment assessment. Regarding dosage of treatment, since the number of sessions in the face-to-face parent training and the number of online training parts in the blended parent training did not correspond one-to-one, we calculated dosage by counting the number of completed training topics: introduction, psychoeducation, ABC charts, recording of observations and impeding factors, communication, offering structure, setting rules, rewarding, ignoring and punishing, reward system, and time out. We explored the relation between treatment dosage and disruptive behavior improvement by correlating dosage and improvement on HSQ-PDD and ABC Irritability within each parent training condition.

Our comparison showed that self-rated therapist fidelity was high in both conditions: therapists rated their fidelity with the face-to-face parent training 93.8% (*n* = 33); for the blended parent training, this was 89.5% (*n* = 27). With regard to premature discontinuation of the face-to-face parent training, 6.1% of parents dropped out in the first phase of parent training, 6.1% in the second, and 9.1% in the third (percentages of total *n* = 33; see Table 1 for the four phases). In contrast, many parents in the blended parent training group stopped the training prematurely and many of them did so early in the training: 54.5% dropped out in the first phase, 24.2% in the second, and 6.1% in the third (percentages of total *n* = 33). A chi-square test showed that the phase when parents stopped differed significantly between the two parent training formats (χ^2^ = 30.83, *p* < 0.001).

For parents in the face-to-face condition, not completing parent training was due to external circumstances (e.g., moving, parental illness), parents preferring help directly for the child, more intensive care being indicated by the clinician, and disruptive behaviors being no longer troublesome. Discontinuation of the blended training was due to parents finding the training too time-consuming, preferring face-to-face contacts, disruptive behaviors being no longer troublesome, parents having difficulty to motivate themselves to work in the program, the clinician indicating more intensive care, and external circumstances.

Therapists added few extra sessions in both training formats. For the 33 participants receiving face-to-face training, additional sessions were not provided in 60.6% of the cases, 21.2% of participants had one extra session, 15.2% had two extra sessions, and 3.0% received three extra sessions. In the blended parent training, of the 33 participants, 63.6% received no extra face-to-face sessions, 24.2% received one, 9.1% received two, and 3.0% received one extra session. Numbers of extra face-to-face sessions were similar for completers and non-completers in the blended condition. In the blended condition, 80.0% of the 5 completers did not receive an extra session and 20.0% received one extra session. Of the 28 non-completers, most (60.7%) received no extra face-to-face sessions, 25.0% received one, 10.7% received two, and 3.6% received three extra sessions, thus the largest part of treatment was still provided online.

Finally, our exploratory analyses of the effect of treatment dosage on improvement on HSQ-PDD and ABC Irritability showed no significant effect in the face-to-face condition, nor in the blended condition.

## Discussion

The findings of the current study indicate that face-to-face behavioral parent training is effective in reducing disruptive behaviors (i.e., noncompliance and irritability) in 4- to 13-year-old children with ASD in routine mental health care, compared to waitlist. Furthermore, our findings suggest that changes in disruptive behaviors sustain up to 6 months after the treatment has ended. We did not demonstrate the effectiveness of the blended (i.e., therapist-assisted online) format of the same training as compared to waitlist. Notably, for the blended format, the level of treatment dropout was very high.

Our findings extend the results of previous studies that showed the efficacy (Postorino et al., 2017; Tarver et al., 2019) of behavioral parent training programs for ASD under more controlled conditions than in routine mental health care. Conducting studies in routine mental health care is relevant because the results have a higher ecological validity and can thus be better generalized to other clinical care settings than studies that are conducted under more controlled circumstances. The medium effect sizes of the face-to-face parent training (i.e., 0.53 for noncompliance and 0.62 for irritability) correspond to effect sizes that have been found in meta-analyses (Postorino et al., 2017; Tarver et al., 2019), even though our effect size for noncompliance should be interpreted in the light of the interaction effect found between age and treatment condition.

In the face-to-face condition, as many as 78.1% of children improved 25% or more over time on noncompliance, which has been described as clinical improvement (Bearss et al., 2015). On average, children’s noncompliance reduced with 47.3% in the face-to-face condition, which is somewhat less than found in two comparable studies, i.e., 55.0% (Bearss et al., 2015) and 71.5% (Aman et al., 2009). The higher percentages in these previous studies might stem from the better controlled circumstances than routine mental health care or from differences in samples (recruited vs referred). Greater mean improvement in other studies may also have arisen from these studies studying a more severe population. Relatedly, and in correspondence to results of other studies (Farmer et al., 2012; Lundahl et al., 2006), we found that a higher level of disruptive behavior at baseline was associated with more improvement over time.

Our findings suggest that the effect of face-to-face parent training on noncompliance, in comparison to having to wait, might be especially evident in younger children. However, our sensitivity analysis of the HSQ-PDD model (in which outliers were excluded) showed a positive age effect on noncompliance improvement regardless of parents receiving parent training or having to wait. Similar to the latter, older children showed greater improvement on irritability, independent of parents having received training or having to wait. Results of former studies into prediction or moderation effects of age are inconsistent. It has been suggested that older children might improve more over time on noncompliance than younger children (Farmer et al., 2012), while other studies suggested a relatively large effect of parent training on disruptive behaviors in a young age group (Tarver et al., 2019), or alternatively, did not find an age effect (Lundahl et al., 2006). While more research into the effect of age is warranted, it appears that offering face-to-face parent training can be a valuable intervention strategy for younger as well as older children with ASD and disruptive behaviors.

To the best of our knowledge, this was the first study to investigate the effect of a blended parent training on disruptive behaviors in children with ASD compared to a waitlist control group. Although the high level of treatment dropout obstructs drawing conclusions about the effect of a completed blended training, including all participants (dropouts and completers) in our analyses renders valuable information for clinical care, pointing to possible difficulties in implementing blended parent training for children with ASD. The high level of treatment dropout in the blended training is a relevant finding in itself and is especially relevant since parenting interventions for ASD are increasingly being offered in other formats than face-to-face (Bearss et al., 2018; Blackman et al., 2020; Ibañez et al., 2018; Ingersoll et al., 2016; Kuravackel et al., 2018; Pacione, 2022). Furthermore, it is important to note that attrition may even be higher outside of a controlled clinical trial in which the motivation to complete the training might be influenced by participating in a clinical study (Ingersoll et al., 2017).The high level of attrition in our blended condition is in line with the level of attrition in a previous study of the same blended parent training in children with ADHD (Breider et al., 2019) and with the high level of attrition found in a web-based training for parents of children with ASD and challenging behaviors (Turgeon et al., 2021). However, our results stand in contrast with studies demonstrating the effect of completely or partially online parent training programs targeting behavioral problems in general population samples (Thongseiratch et al., 2020) or targeting other domains of functioning of children with ASD, such as interaction and communication (Blackman et al., 2020; Ibañez et al., 2018; Ingersoll et al., 2016), executive functioning (Kenworthy et al., 2023), toilet training (Little et al., 2023), or sleep (Roberts et al., 2019). Note, however, that in the studies of Kenworthy et al. (2023) and Little et al. (2023) parents used the online materials less compared to direct contacts with a therapist, similar to the findings in our study.

An explanation for the high attrition level in our study may be that, due to the high family burden related to child ASD and disruptive behaviors, the online parts of a blended training demand too much time and independent effort of parents of children with ASD and disruptive behaviors (Herring et al., 2006; Huang et al., 2014; Sikora et al., 2013). Relatedly, an often-stated reason for discontinuing the blended parent training in our study was the time investment that the training demanded. It might also be that parents are more motivated to address developmental challenges in their child with ASD, such as communication and social interaction (e.g., Blackman et al., 2020), as they may perceive these as more beneficial.

Although research has shown that parents value accessible and flexible training programs, such as online training formats (Raulston et al., 2019; Ros-DeMarize et al., 2023), the parents in our study seemed to have trouble to fit in the opportunity to work in the online program, while other pressing daily issues also demanded their attention. To overcome barriers in working in the online program, in the future brief parent training programs (e.g., Hornstra et al., 2021) might be of value to increase the accessibility of parent training for those parents. In addition, it may have been that the less structured blended training, as compared to the face-to-face training, did not entirely match parents’ needs. Importantly, it may be that the needs of parents differ substantially across parents, for example due to the level of disruptive behaviors of their child, parental psychopathology (Ros-DeMarize et al., 2023), parental age (Gentile et al., 2022), or busy schedules. Parents’ specific needs should possibly be addressed before the start of parent training to optimize treatment engagement.

Also contrasting to our findings regarding the blended parent training are the positive results of studies into parent training for disruptive behaviors in children with ASD by videoconference (Bearss et al., 2018; Kuravackel et al., 2018; Martin et al., 2023). Videoconference appears to be the main method used in online interventions directed at parents of children with ASD and the evidence base for videoconference interventions is far larger than interventions using online training modules as in our study (Ellison et al., 2021). However, videoconference appears to be more similar to face-to-face parent training than to blended parent training: during videoconference parents have direct (real-time) contact with the therapist. The mode of transfer of knowledge and information is thus similar to face-to-face training and parents might experience this form of help as more structured and tailored to their needs. Furthermore, online provision of real-time parent training might even improve ecological validity by planning sessions at the actual times that problems arise (morning routine, meal routine, etc.; Sullivan et al., 2021). Also, some of the videoconference parent training programs were provided during the COVID pandemic (e.g., Martin et al., 2023), a period in which, in many countries, face to face parent training could not be provided.

Therapist contact has been shown to have a positive effect on online parent training (Day & Sanders, 2018; Ingersoll et al., 2016; Tarver et al., 2014) and a meta-analysis studying online parent training for behavioral problems (although in non-ASD samples) found that sending parents reminders to work on the program contributed to higher effectiveness (Thongseiratch et al., 2020). Although the blended training in our study included multiple face-to-face sessions and parents were sent a reminder if they did not work in the program for two weeks, perhaps, the amount of direct therapist contact (i.e., speaking to or seeing the therapist) in our blended training was not sufficient to meet the parent’s needs. This is in line with multiple parents in our study indicating that they discontinued the blended parent training because they preferred face-to-face contacts. Future research should study if blended parent training for children with ASD and disruptive behaviors benefits from more direct face-to-face contact (either on location or through video-conference) and, if so, what would then be the ideal balance.

The finding that many parents in the blended condition discontinued early on in the training, i.e., before the primary intervention content was presented, also gives lead to investigate whether sooner introduction of this content would improve the attendance and effect (Ingersoll et al., 2017). Perhaps, faster steps can be made in the first three modules of the training, for example by offering this content in a more structured way, and face-to-face (either office-based or by videoconference) with a therapist. This might also help to strengthen the therapeutic alliance, and consequently, engagement with the training (Hock et al., 2015; Mohr et al., 2011).

In the face-to-face condition, our findings showed sustained improvement in disruptive behaviors from post-treatment to 6 months follow-up, suggesting long term changes after face-to-face parent training, which is in line with other studies (Bearss et al., 2015; Sofronoff et al., 2004; Tellegen & Sanders, 2014; Whittingham et al., 2009). Although participants were allowed to receive other health care during the follow-up period, our findings are not likely to be affected by additional care after the training, since excluding participants who received additional health care in the follow-up period did not change the outcomes.

### Strengths and Limitations

This study adds to the existing literature by investigating two formats of behavioral parent training for ASD in routine mental health care. The study is limited in that we did not achieve our intended sample size. Perhaps, in a larger sample, the blended training would have shown an effect on reducing irritability, as the regression coefficient approached significance and there seemed to be some reduction in irritability over time. However, the high amount of (early) attrition in the blended condition is an important, clinically relevant finding of the current study and we have no indication to assume that this would have been different in a larger sample. In the current study, since there were only five completers, we did not test the effect of the blended parent training in completers only. In future studies, it may be interesting to test this and to examine what kind of parents complete a blended training. Furthermore, our study was conducted before the COVID-19 pandemic, while currently parents and therapists may be more used to (partially) online treatment. It would be interesting to examine the effect of a blended parent training again in a time where e-health is more usual.

Another limitation of the study is that the children in our sample were intellectually relatively high functioning, had relatively low levels of disruptive behaviors, and had parents with a relatively high educational level, in comparison to other studies. It could be that parents of children with lower IQs and more severe problems, and with a lower educational level were less willing to be randomized. Furthermore, although we did not test this, it may have been that the effects of parent training on noncompliance were larger for the daily home situations that were targeted in the training, as compared to the main effects. Therefore, in future studies, it may be of value to compare the effects of the training in targeted versus non-targeted situations. Such a comparison might also yield valuable insights into the generalization of improvements. A limitation regarding our outcome measures is that we did not use masked measures to assess child behavior. However, the use of questionnaires corresponds to measurement methods in clinical care. Furthermore, we used a self-rated therapist checklist to measure treatment integrity, instead of a more objective measure, such as audio records rated by masked researchers. Finally, because our study did not contain a 6 months follow-up in the waitlist control condition and parents were allowed to use other health care in the follow-up period, definite conclusions regarding long-term effectiveness could not be drawn.

### Clinical Implications

Our results encourage the use of face-to-face behavioral parent training in routine mental health care to reduce disruptive behaviors, specifically noncompliance and irritability, in children with ASD without an intellectual disability who show relatively mild disruptive behaviors. Our findings suggest that the face-to-face training may be helpful for children of a wide age range. Furthermore, children improved independent of their sex, intelligence, ASD severity level, and the educational level of their parents. So far, our findings do not advocate the use of our blended parent training for children with ASD and disruptive behaviors. Adapting the blended format, for example through increasing real-time therapist contact, might be an option for strengthening therapeutic alliance, reducing attrition, and increasing effects. Alternatively, it could be that blended parent training is more suitable for other populations, such as children not in clinical (referred) care. Since research into e-health interventions for children with ASD and disruptive behaviors mainly focuses on videoconference interventions, we welcome future studies into (blended) programs using online training modules, as well as studies focusing on the most optimal format and the population who benefits from such programs. For referred children with ASD and disruptive behaviors, the face-to-face parent training format currently appears to be the format of preference.

## Supplementary Information

Below is the link to the electronic supplementary material.

Supplementary file 1 (DOCX 21 KB)

Supplementary file 2 (DOCX 36 KB)

Supplementary file 3 (DOCX 78 KB)

## Data Availability

Authors are willing to send relevant documentation or data upon request, if in line with informed consent given by participants.
