# Supplementary material for: Parent Training for Disruptive Behaviors in Referred Children with Autism Spectrum Disorder: A Randomized Controlled Trial
Source: J Autism Dev Disord. 2024 Sep 27;56(2):481–98. doi: 10.1007/s10803-024-06567-0 (PMC12864344; doi:10.1007/s10803-024-06567-0)
Supplement: Supplementary file 1 — (DOCX 21 KB) [file 10803_2024_6567_MOESM1_ESM.docx]

**Supplementary file 1**

Items of the list of target behaviors

| Item |
| --- |
| 1. Noncompliance (not following rules / not doing what parents say) |
| 2. Whining / nagging / disagreeing / discussing |
| 3. Challenging / provoking / intentionally annoying others |
| 4. Being angry quickly and often |
| 5. A lot of arguing with siblings |
| 6. Temper tantrums |
| 7. Swearing |
| 8. Hitting / pinching / kicking |
| 9. Breaking items / throwing items |
| 10. Losing things / forgetting things / being chaotic |
| 11. Not cleaning up things |
| 12. Not completing tasks |
| 13. Not being able to wait for his / her turn |
| 14. Running away / being difficult to control |
| 15. Overactive in talking (talking a lot / skip from one subject to another) |
| 16. Being loud / making noise |
| 17. Claiming attention |
| 18. Acting as "a clown" |
| 19. Interfering with others a lot / interrupting others |
| 20. Claiming of father or mother / not being able to do anything without them |
| 21. Not being able to play alone |
| 22. Does not understand instructions / assignments |
| 23. Cannot be deterred from own idea / desire |
| 24. Must do something in constantly exactly the same way |
| 25. Is constantly busy with specific subject(s) |
| 26. Asking questions repetitively (repeating questions over and over again) |
| 27. Resistance and / or panic in response to changes / transitions / unexpected situations |
| 28. (Unpredictable) anxious behavior |
| 29. Does dangerous things |
| 30. Hurting themselves / automutilation |
| 31. Talks / thinks negatively about themselves |
| 32. Is afraid of doing things wrong |

*Note*. Items of the list of target behaviors (Van den Hoofdakker et al., 2007) were translated by

the researchers for reference purposes, i.e., this is not an official English translation of the list.

Parent Training for Disruptive Behaviors in Referred Children with Autism Spectrum Disorder: A Randomized Controlled Trial. Journal of Autism and Developmental Disorders. Simone Breider, Annelies de Bildt, Kirstin Greaves‑Lord, Andrea Dietrich, Pieter J. Hoekstra, Barbara J. van den Hoofdakker. Corresponding author: a.de.bildt@accare.nl.
