# Supplementary material for: Parent Training for Disruptive Behaviors in Referred Children with Autism Spectrum Disorder: A Randomized Controlled Trial
Source: J Autism Dev Disord. 2024 Sep 27;56(2):481–98. doi: 10.1007/s10803-024-06567-0 (PMC12864344; doi:10.1007/s10803-024-06567-0)
Supplement: Supplementary file 2 — (DOCX 36 KB) [file 10803_2024_6567_MOESM2_ESM.docx]

**Supplementary file 2**

**
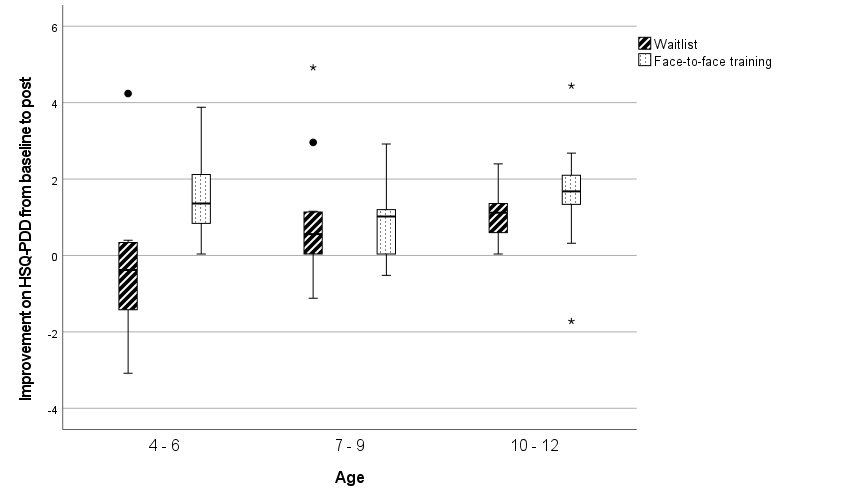
**

Boxplot of age by HSQ-PDD improvement from baseline to post-treatment (raw scores) for the waitlist and face-to-face training condition for three age categories

Parent Training for Disruptive Behaviors in Referred Children with Autism Spectrum Disorder: A Randomized Controlled Trial. Journal of Autism and Developmental Disorders. Simone Breider, Annelies de Bildt, Kirstin Greaves‑Lord, Andrea Dietrich, Pieter J. Hoekstra, Barbara J. van den Hoofdakker. Corresponding author: a.de.bildt@accare.nl.
