# Supplementary material for: Parent Training for Disruptive Behaviors in Referred Children with Autism Spectrum Disorder: A Randomized Controlled Trial
Source: J Autism Dev Disord. 2024 Sep 27;56(2):481–98. doi: 10.1007/s10803-024-06567-0 (PMC12864344; doi:10.1007/s10803-024-06567-0)
Supplement: Supplementary file 3 — (DOCX 78 KB) [file 10803_2024_6567_MOESM3_ESM.docx]

**Supplementary file 3**

**
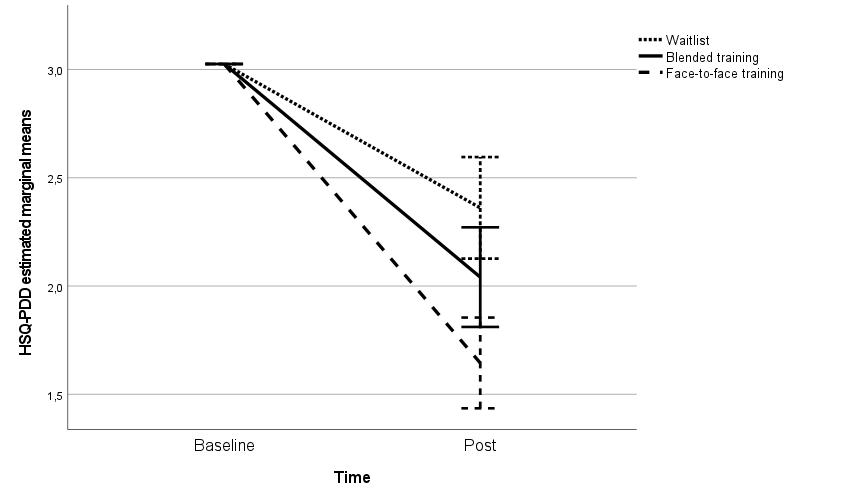
**

**a**

**
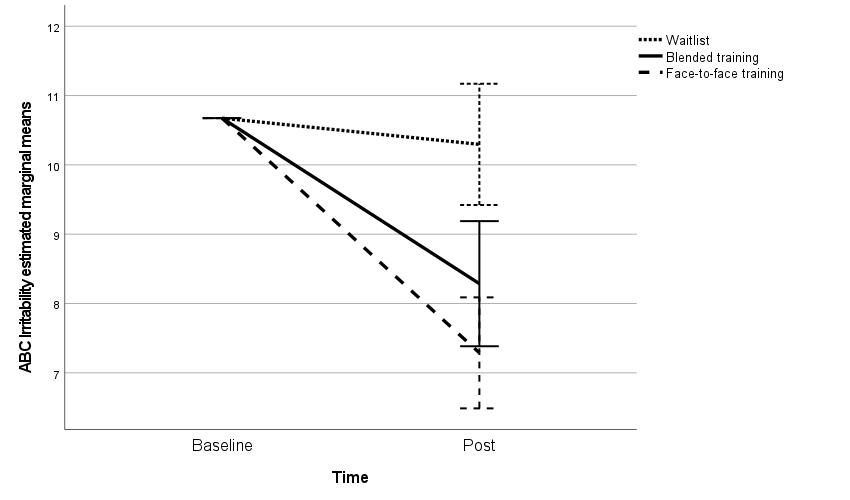
**

b

Plots of HSQ-PDD (a) and ABC Irritability (b) estimated marginal means (corrected for baseline score and age) at baseline and post-treatment per condition, with 1 SE error bars to display variability of outcome

Parent Training for Disruptive Behaviors in Referred Children with Autism Spectrum Disorder: A Randomized Controlled Trial. Journal of Autism and Developmental Disorders. Simone Breider, Annelies de Bildt, Kirstin Greaves‑Lord, Andrea Dietrich, Pieter J. Hoekstra, Barbara J. van den Hoofdakker. Corresponding author: a.de.bildt@accare.nl.
